# Influences of hand dominance on the maintenance of benefits after home-based modified constraint-induced movement therapy in individuals with stroke

**DOI:** 10.1590/bjpt-rbf.2014.0050

**Published:** 2014

**Authors:** Renata C. M. Lima, Lucas R. Nascimento, Stella M. Michaelsen, Janaine C. Polese, Natália D. Pereira, Luci F. Teixeira-Salmela

**Affiliations:** 1 Departamento de Fisioterapia, Universidade Federal de Minas Gerais (UFMG), Belo Horizonte, MG, Brasil; 2 Centro Universitário Newton Paiva, Belo Horizonte, MG, Brasil; 3 Faculty of Health Sciences, The University of Sydney, Sydney, NSW, Australia; 4 Departamento de Fisioterapia, Universidade do Estado de Santa Catarina (UDESC), Florianópolis, SC, Brasil

**Keywords:** cerebrovascular disease, hemiparesis, upper extremity, rehabilitation, hand dominance

## Abstract

**Objective:**

To investigate the influence of hand dominance on the maintenance of gains after home-based modified constraint-induced movement therapy (mCIMT).

**Method:**

Aprevious randomized controlled trial was conducted to examine the addition of trunk restraint to the mCIMT. Twenty-two chronic stroke survivors with mild to moderate motor impairments received individual home-based mCIMT with or without trunk restraints, five times per week, three hours daily over two weeks. In this study, the participants were separated into dominant group, which had their paretic upper limb as dominant before the stroke (n=8), and non-dominant group (n=14) for analyses. The ability to perform unimanual tasks was measured by the Wolf Motor Function Test (WMFT) and the Motor Activity Log *(*MAL), whereas the capacity to perform bimanual tasks was measured using the Bilateral Activity Assessment Scale (BAAS).

**Results:**

Analysis revealed significant positive effects on the MAL amount of use and quality of the movement scales, as well as on the BAAS scores after intervention, with no differences between groups. Both groups maintained the bimanual improvements during follow-ups (BAAS-seconds 0.1, 95% CI -10.0 to 10.0), however only the dominant group maintained the unilateral improvements (MAL-amount of use: 1.5, 95% CI 0.7 to 2.3; MAL-quality: 1.3, 95% CI 0.5 to 2.1).

**Conclusions:**

Upper limb dominance did not interfere with the acquisition of upper limb skills after mCIMT. However, the participants whose paretic upper limb was dominant demonstrated better abilities to maintain the unilateral gains. The bilateral improvements were maintained, regardless of upper limb dominance.

## Introduction

Stroke is the leading cause of adult disabilities worldwide^1^. Four out of five stroke survivors experience acute upper limb weaknesses and between 45% and 75% of the patients continue to have limited upper limb function six months after stroke^2^^,^ 3. According to Taub et al.^4^, many patients use their nonparetic upper limb to perform their daily activities, which progressively decrease the amount and quality of use of the paretic upper limb. This learned non-use phenomenon is responsible for increased weaknesses, decreased abilities of the paretic upper limb in performing unimanual and bimanual activities, and restricted social participation^5^^-^ 9. Therefore, the treatment of such residual deficits is critically important for the stroke population and Constraint-Induced Movement Therapy (CIMT) has emerged as a promising intervention to improve upper limb function after stroke^10^^,^ 11. 

Originally, the therapy was delivered for six hours a day over two weeks, but current evidence suggested modifications to the protocol to improve the efficacy and efficiency of the intervention regarding the patients'needs and preferences^4^^,^ 11. Nowadays, CIMT is clearly described as a behavioral intervention, which allows patients to actively explore new possibilities of actions and should be composed of three pillars: (i) intensive and repetitive task-oriented training of the paretic upper limb, following the principles of difficulty in the progression and involvement of functional training, carried out by shaping and task practices; (ii) the transfer package, which includes a set of behavioral methods to transfer the gains of supervised training to the individuals' real world; and (iii) the restriction of the non-paretic upper limb during 90% of awake hours, during the training days^12^^,^ 13. 

Most of the studies that investigated the effects of CIMT have demonstrated significant gains in upper limb function, as well as increases in the paretic upper limb use during daily activities^11^^,^ 14 ^,^ 15. Some of these studies also reported the long-term effects of the CIMT, suggesting that the benefits were maintained up to two years^14^^,^ 16. A large randomized clinical trial with 222 participants found that, from baseline to 12 months, the CIMT group showed greater improvement regarding performance and the amount and quality of use of the paretic upper limb, compared with controls^15^. Although the short- and long-term benefits of CIMT have already been described, it is well known that most individuals have one upper limb which performs daily skills more proficiently^17^^,^ 18. Thus, it is possible that upper limb dominance prior to stroke may interfere with the acquisition and the maintenance of upper limb skills, due to the specific brain activation patterns or the amount of upper limb use during daily activities. 

The immediate influences of upper limb dominance after CIMT were examined only in one study with nine individuals after stroke and no significant differences were found between the participants who had their paretic upper limb as dominant, compared to those who had the non-paretic upper limb as dominant^17^. However, improvements associated with the intervention were observed for both groups. These results suggested that dominance may not interfere with the acquisition of upper limb skills after an intensive unimanual intervention approach, but provided no information regarding the influences of upper limb dominance on the maintenance of the acquired skills. 

Thus, the aim of the present study was to compare the immediate effects and the maintenance of the effects after a home-based modified CIMT (mCMIT) on measures of upper limb capacity and performance of individuals with chronic stroke, taking into account their previous upper limb dominance, to better comprehend the influences of upper limb dominance associated with this therapy. 

## Method

### Design

**Figure 1:**
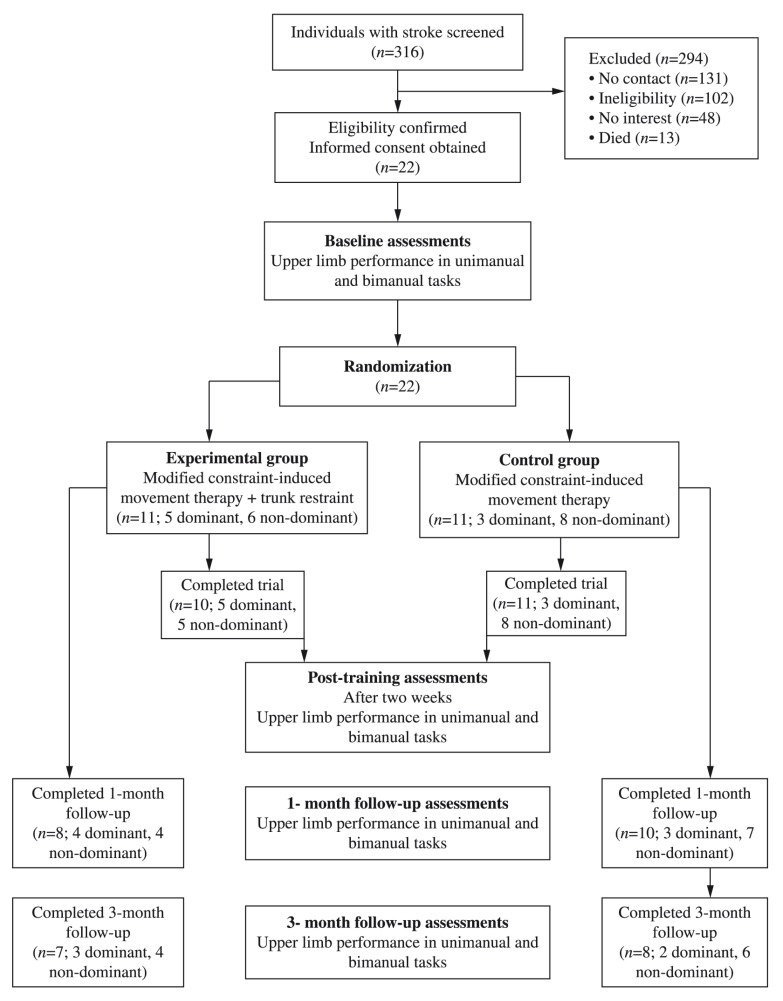
Flow of participants through the trial.

This study followed the design previously described regarding the protocol of a randomized clinical trial^12 ^(Figure 1). The study was registered and allocated by the Australian New Zealand Clinical Trials Registry-ACTRN (ACTRN12610000698077) and obtained ethical approval from the Human Research Ethical Committee (#0408·0-203·000-09) of Universidade Federal de Minas Gerais (UFMG), Belo Horizonte, Brazil. All participants signed an informed consent form. 

Data from 22 participants who had completed the two-week intervention of home-based mCIMT with and without trunk restraint were analyzed to investigate the influences of hand dominance on the acquisition and maintenance of the improvements. Since no differences were found between the groups (with and without trunk restraint), the present study included all subjects and took into account only the hand dominance prior to stroke. For analyses, the participants were separated into two groups: Dominant, for subjects whose paretic upper limb was dominant before the stroke, and non-dominant, for subjects whose paretic upper limb was non-dominant before the stroke. Hand dominance was reported by the individuals when asked which hand they used to write a sentence. The severity of the motor impairments was determined based upon the upper limb items of the Brazilian version of the Fugl-Meyer scale, with mild scores ranging from 51 to 66, and moderate ones, from 26 to 50^19^. 

### Participants

Chronic stroke survivors were recruited from the general community of the city of Belo Horizonte, Brazil, and were considered eligible if they met the inclusion and exclusion criteria established according to the protocol of this randomized clinical trial^12^. 

### Intervention

All participants received individual home-based mCIMT with or without trunk restraints from well-trained physical therapists, five times per week, three hours daily over two weeks. They were encouraged to use a glove to restrict their non-paretic wrist and fingers for 90% of the time that they were awake during the two-week period. A diary was provided to all participants to record the amount of time they wore the glove. Three-hour sessions of mCIMT included 30 minutes of the transfer package exposure and applications of the items of the Motor Activity Log (MAL)^20^^,^ 21, followed by two hours and 30 minutes of four shaping tasks, and one complete practice task. Task difficulty was individually adjusted to be sufficiently challenging, as determined by the physical therapist, and shaping techniques were incorporated, with increasing task difficulty over successive sessions. Blood pressure measures were obtained before and after the interventions, and heart rates were continuously monitored by a heart rate monitor (Polar, modelo S 610i)^2^^,^ 12. 

### Primary outcome measures

The primary outcome measures included upper limb performance, as determined by the MAL^20^^,^ 21, and upper limb capacity, as measured by the Wolf Motor Function Test (WMFT)^22^^,^ 23. 

The MAL was developed to evaluate the effects of CIMT. This scale, adapted to the Portuguese-Brazilian language, contains 30 items related to routine daily activities undertaken with the paretic upper limb. The individuals were asked about the amount of use (AOU) and the quality of movement (QOM) with their paretic upper limb during their daily activities. The MAL total scores were obtained by the sum of the answers divided by the number of the assessed items, which ranged from five to zero. Higher scores are indicative of better performance of the paretic upper limb^20^^,^ 21. 

Out of the 17 WMFT tasks, 15 were timed, and the maximum time allowed for the completion of each task was 120 seconds, which was qualified in accordance with these scores. The test was always initiated with the examiner explaining and demonstrating the execution of all of the tasks at both the slow and fast speeds. The evaluations were videotaped, so that the functional ability, or the qualitative scores, was also analyzed after the completion of the evaluations. All subjects were allowed to perform the tasks with their non-paretic upper limb first for familiarization purposes^22^^,^ 23. 

### Secondary outcome measures

Secondary outcomes included the speed and the quality of the paretic upper limb use during bimanual tasks, as determined by the Bilateral Activity Assessment Scale (BAAS) scores. The BAAS was developed to evaluate the interactions between the paretic and non-paretic upper limb during the performance of 13 bimanual activities. All activities were timed and the maximum time allowed for the completion of each task was 120 seconds. The evaluations were also videotaped, so that the functional ability scores or the qualitative scores were also analyzed after the completion of the evaluations. The test was always initiated with the examiner explaining and demonstrating the execution of all of the tasks, but no information regarding the speed of execution was provided^24^^,^ 25. The evaluations of the subjects'performances were carried out by a physical therapist, who was blinded the group and the time of the evaluations. 

### Data analysis

Database management and statistical analyses were performed by an independent researcher, who was blinded to the group allocations. All measures were analyzed with intention-to-treat analyses, and descriptive statistics were calculated for all outcome measures. Analyses of covariance (ANCOVA), which controlled for the baseline characteristics, were employed to analyze the effects of the intervention. The results were reported as means and standard deviations or means and 95% confidence intervals (CI). Repeated-measures ANOVA, followed by pre-planned contrasts, were used to verify the main and interaction effects within and between groups for the four time points. To better understand the influences of upper limb dominance on the acquisition and maintenance of the improvements, the differences between the groups were provided as means and 95% CI. This type of analysis was chosen because, while the null hypothesis significance tests use probability levels (e.g. p<0.5), effect size analyses focus on the magnitude of the differences between the groups and the probability of an effect to report and interpret the results. This type of description assists in determining the clinical interpretation and importance of the observed differences, as well as the statistical significance of the findings^26^^,^ 27. All analyses were performed with SPSS, version 17.0 for Windows. 

## Results

### Participants

Twenty-two individuals with chronic unilateral stroke, who had a mean time since the onset of stroke of 81±49 months participated. All participants were right-hand dominant prior to stroke. Eight subjects had left hemispheric stroke (mean age of 58±11 years; mean time since stroke of 89±35 months; and mean Fugl-Meyer UL scores of 50±6). Fourteen participants had right hemispheric stroke (mean age of 59±7 years; mean time since stroke of 76±56 months; and mean Fugl-Meyer UL scores of 46±9). The participants' characteristics are described in Table 1. 

**Table 1:** Baseline characteristics of the participants and comparison between the groups (statistical tests and p values).

Characteristics	Groups		Comparison between groups
	Dominant (*n*=8)	Non-dominant (*n*=14)	Statistical tests, *p* values
Age (years), mean (SD)	58 (11)	59 (7)	*t *=1.22; *p *=0.75
Gender, *n* men (%)	4 (50)	7 (50)	*X * ^*2 *^=0.0; *p *=1.0
Dominant side, n Right (%)	8 (100)	14 (100)	*X * ^*2 *^=0.01; *p *=0.99
Time since the stroke (months), mean (SD)	89.0 (35.0)	76.0 (56.0)	*t *=1.84; *p *=0.54
Cognition (MMSE 0-30), mean (SD)	26 (3.5)	25 (3.5)	*U *=0.52; *p *=0.61
Motor impairments (Fugl-Meyer upper limb scores: 0-66), mean (SD)	50 (6)	46 (9)	*U *=1.3; *p *=0.19
Mean upper limb muscle strength (Nm), paretic side, mean (SD)	48.6 (26.7)	52.0 (23.2)	*t *=0.05; *p *=0.54
Mean upper limb muscle strength (Nm), non-paretic, mean (SD)	68.7 (42.0)	69.9 (27.0)	*t *=4.31; *p *=0.93
Elbow muscle tone *(*Modified Ashworth scores: 0-4, *n* (%)			*X * ^*2 *^=4.61; *p *=0.33
0	3 (37.5)	2 (14.3)	
1	2 (25.0)	1 (7.1)	
1+	1 (12.5)	1 (7.1)	
2	1 (12.5)	6 (42.9)	
3	1 (12.5)	4 (28.6)	
Wrist muscle tone *(*Modified Ashworth scores: 0-4, *n* (%)			*X * ^2^=7.76; *p *=0.1
0	7 (87.5)	4 (28.6)	
1	0	2 (14.3)	
1+	1 (12.5)	3 (21.4)	
2	0	2 (14.3)	
3	0	3 (21.4)	

MMSE = Mini-mental state examination

t =Student t-test for independent samples

X2 = Chi-square

U = Mann-Whitney U

### Immediate effects of the intervention

As shown in Table 2, significant gains after the home-based mCIMT were observed for both groups regarding their MAL, AOU, and QOM scores, and the time required to perform bimanual tasks. There were no differences between the groups immediately after the intervention, suggesting that upper limb dominance did not interfere with the acquisition of the upper limb skills. Although both groups improved the mean time to perform unilateral tasks, as determined by the WMFT scores, changes were significant only for the non-dominant group. No significant gains were observed regarding the QOM for either unimanual or bimanual tasks, indicating that the interventions were neither beneficial nor detrimental regarding the movement patterns (Table 2). 

**Table 2:** Means (SD) of the outcome measures at baseline and post-training, means (95% CI) of the within- and between-group differences, and ANCOVA statistics.

Outcome	Groups			
	Week 0		Week 2	
	Dominant (*n*=8)	Non-dominant (*n*=14)	Dominant (*n*=8)	Non-dominant (*n*=14)
MAL, *amount of use* (0-5)	1.5 (0.7)	0.7 (0.4)	3.5 (0.7)	2.5 (0.8)
MAL, *quality of movement* (0-5)	1.6 (0.9)	0.6 (0.5)	3.0 (0.8)	2.4 (0.7)
WMFT (seconds)	16.8 (20.0)	15.8 (11.5)	11.2 (11.9)	11.7 (9.9)
WMFT, *quality of movement* (0-5)	3.1 (0.5)	2.6 (0.6)	3.2 (0.4)	2.8 (0.7)
BAAS (seconds)	38.3 (12.9)	38.1 (12.1)	33.8 (13.0)	32.1 (11.4)
BAAS, *quality of movement* (0-65)	40.0 (10.0)	28.7 (12.5)	41.1 (9.9)	31.3 (12.4)
** Outcome **	**Difference within groups**		**Difference between groups**	
	**Week 2 minus Week 0**		**Week 2 minus Week 0**	**Week 2 measurements**
	**Dominant**	**Non-dominant**	**Dominant minus non-dominant**	**F; p**
MAL, *amount of use* (0-5)	2.0 (1.4 to 2.6)	1.8 (1.3 to 2.2)	0.6 (–0.2 to 1.5)	*F * =2.2; *p= * 0.1
MAL, *quality of movement* (0-5)	1.4 (0.7 to 2.2)	1.8 (1.3 to 2.2)	0.3 (–0.5 to 1.1)	*F * =0.6; *p= * 0.5
WMFT (seconds)	–5.6 (–15.0 to 3.9)	–4.0 (–7.4 to –0.7)	–1.1 (–6.4 to 4.1)	*F * =0.2; *p= * 0.6
WMFT, *quality of movement* (0-5)	0.1 (–0.2 to 0.5)	0.2 (–0.1 to 0.4)	0.02 (–0.4 to 0.4)	*F * =0.1; *p= * 0.9
BAAS (seconds)	–4.5 (–6.3 to –2.7)	–6.0 (–9.5 to –2.4)	–1.4 (–6.2 to 3.4)	*F * =0.4; *p= * 0.5
BAAS, *quality of movement* (0-65)	1.1 (–2.4 to 4.7)	2.6 (–0.1 to 5.3)	0.5 (–4.2 to 5.2)	*F * =0.1; *p= * 0.8

MAL = Motor Activity Log

WMFT = Wolf Motor Function Test

BAAS = Bilateral Activity Assessment Scale

### Maintenance of the effects of the intervention

The follow-up comparisons between the groups demonstrated that, although similar improvements were observed for both groups immediately after the intervention, only the participants of the dominant group were able to retain the achieved gains regarding the MAL scores. The time required to perform unimanual or bimanual tasks did not differ between the groups with the follow-up measurements and the bimanual gains were maintained for both groups (Table 3). 

**Table 3:** Means (SD) of the outcome measures at one- and three-month follow-ups, means (95% CI) of the within- and between-group differences.

Outcome	Groups				Difference within groups				Difference between groups	
	1-month follow-up		3-month follow-up		1 –month follow-up		3-month follow up		1-month follow-up	3-month follow-up
	Dominant (*n*=8)	Non-dominant (*n*=14)	Dominant (*n*=8)	Non-dominant (*n*=14)	Dominant (FU1 minus post)	Non-dominant (FU1 minus post)	Dominant (FU3 minus post)	Non-dominant (FU3 minus post)	Dominant minus non-dominant	Dominant minus Non-dominant
MAL, *amount of use* (0-5)	3.4 (0.6)	2.1 (0.8)	3.4 (0.7)	1.9 (0.9)	–0.1 (–0.3 to 0.2)	–0.3 (–0.5 to –0.1)	–0.1 (–0.8 to 0.6)	–0.5 (–0.9 to –0.2)	1.3 (0.6 to 2.0)	1.5 (0.7 to 2.3)
MAL, *quality of movement* (0-5)	3.2 (0.7)	2.1 (0.7)	3.2 (0.7)	1.9 (0.9)	0.1 (–0.3 to 0.6)	–0.2 (–0.4 to 0)	0.2 (–0.4 to 0.8)	–0.5 (–0.9 to –0.1)	1.1 (0.5 to 1.7)	1.3 (0.5 to 2.1)
WMFT (seconds)	8.9 (8.7)	12.3 (9.9)	8.6 (8.8)	11.6 (9.0)	–2.3 (–7.7 to 3.1)	0.6 (–1.7 to 2.9)	–2.6 (–7.9 to 2.7)	–0.2 (–2.5 to 2.1)	–3.4 (–12.2 to 5.3)	–3.0 (–11.2 to 5.3)
WMFT, *quality of movement* (0-5)	3.2 (0.4)	2.8 (0.7)	3.3 (0.5)	2.8 (0.7)	0 (–0.5 to 0.5)	0 (–0.2 to 0.2)	0.1 (–0.6 to 0.8)	0.1 (–0.2 to 0.1)	0.4 (–0.2 to 1.0)	0.5 (–0.1 to 1.1)
BAAS (seconds)	32.5 (12.1)	33.2 (11.0)	32.6 (12.0)	32.6 (10.2)	–1.3 (–3.5 to 0.9)	1.0 (–2.4 to 4.6)	–1.1 (–3.6 to 1.3)	–0.5 (–2.3 to 3.3)	–0.7 (–11.2 to 9.8)	0 (–10.0 to 10. 0)
BAAS, *quality of movement* (0-65)	43.1 (8.3)	31.2 (12.2)	42.6 (8.6)	32.8 (12.7)	2.0 (–3.3 to 7.3)	–0.1 (–4.1 to 4.0)	1.5 (–3.0 to 6.0)	1.5 (–3.1 to 6.1)	3.3 (–1.1 to 7.8)	1.0 (–3.9 to 6.0)

MAL = Motor Activity Log

WMFT = Wolf Motor Function Test

BAAS = Bilateral Activity Assessment Scale

## Discussion

This was the first study to investigate the influence of upper limb dominance prior to stroke on the acquisition and maintenance of upper limb skills after an intensive unimanual intervention approach. This modality of intervention was chosen due to its characteristics (intensive, repetitive, and progressive practice) and proven efficacy (short- and long-term effects). Although a previous study with a small sample^17^ suggested that upper limb dominance did not interfere with the acquisition of upper limb skills, no information regarding the long-term effects was provided. The results of the present study corroborated other findings and demonstrated that the referred improvements in the AOU and QOM of the paretic upper limb during unimanual tasks, as well as in the time to perform bimanual tasks observed immediately after the mCIMT occurred, regardless of upper limb dominance. However, the follow-up measures indicated that only the participants of the dominant group were able to retain the gains in their unimanual abilities, while both groups maintained their bimanual improvements. These results suggested that upper limb dominance influenced the maintenance of unilateral improvements, but did not affect the maintenance of the gains in bimanual skills. 

The influence of the dominance of the upper limb in carrying out bimanual functional activities was previously evaluated with stroke individuals. McCombe Waller and Whitall^28 ^conducted a training, based upon bimanual activities with 22 individuals, 11 were paretic-side dominant and 11 were pareticside non-dominant. They demonstrated significant training effects for the dominant group^28^. In the present study, the training was specific to the paretic side, regardless of whether it was the dominant one, therefore the dominant and the non-dominant UL received the same training intensity and similar performances were expected for both groups immediately after the intervention. Another issue is that CIMT involves the individuals' commitment as an integral part of the transfer package, because it is an important behavioral therapy imprint^13^. Thus, it was expected that all participants, regardless of upper limb dominance, would demonstrate similar gains, as was found in this study. 

The maintenance of gains appeared to suffer the direct influences of upper limb dominance. This could be explained by the interactions of the individuals in their environment, guided by intrinsic and extrinsic characteristics^29^^-^ 31. Individuals who had past experiences of preferred use of one of the upper limbs prior to the stroke, as was the case of the dominant group, would tend to return to their preferred patterns after the cessation of the intervention. Then the individuals whose paretic side was the dominant side before the stroke would have a greater tendency to maintain gains in unilateral activities, as was evidenced by the maintenance of the MAL scores during the follow-ups only for the dominant group. On the other hand, individuals whose paretic side was the non-dominant side would tend to go back to using their preferred upper limb during unimanual activities as before. Importantly, these results appear to be clinically meaningful. For example, Lang et al.^32 ^demonstrated that improvements of at least 1 point on the MAL scores were considered patient-perceived meaningful changes for stroke participants. This minimal clinically important difference indicates that the between-group differences immediately after the mCIMT were neither statistically, nor clinically significant. However, the observed differences between the dominant and non-dominant groups regarding the maintenance of the gains during the follow-ups proved to be not only statistically, but also clinically, meaningful. Thus, the results demonstrated that, immediately after training, the non-dominant group also showed improvements in upper limb skills regarding the unimanual activities, but these gains were lost over time. 

Although some unilateral gains were lost after the interventions for the non-dominant group, the findings of the present study demonstrated that both groups showed significant improvements in their abilities to execute bimanual tasks immediately after the intervention and that these within-groups gains remained after the cessation of the intervention. These results indicated that intensive unilateral training promoted bimanual ability improvements. Possibly, the achieved gains associated with the intervention were incorporated during the performance of bilateral activities as indicated by the improvements in the time to execute activities measured by the BAAS. The results suggested that the paretic upper limb has the potential to be used after unilateral intervention to help the non-paretic upper limb to execute bilateral activities faster. In addition, the present findings suggested that individuals, regardless of their upper limb dominance, managed to incorporate the immediate gains when performing bimanual activities in their daily routines. These results suggest that performance and capacity for carrying out bimanual tasks are important parameters to measure the real effects of any therapy aimed at improving upper limb activity after stroke. 

The participants of the non-dominant group lost their abilities to perform unimanual tasks after the end of the intervention, but their abilities to perform bimanual tasks were maintained. Since during bimanual tasks they kept the same AOU of their paretic upper limb, this adaptation was integrated into their daily routines, possibly becoming a permanent change. Regarding the maintenance of unimanual gains for the non-dominant group, strategies to monitor losses and avoid the return of the learned disuse phenomenon would be required. These strategies could include periodic revaluations, home guidelines or even more specific interventions, depending upon each case. 

Significant gains in unimanual tasks after training, as evaluated by the WMFT, were found only for the non-dominant group. This could be explained by the small sample size. Considering that the WMFT baseline scores for the dominant group were higher than those of the non-dominant, the sample size may have been insufficient to detect significant changes. For the dominant group, there was a power of 0.12 with an effect size of 0.32, whereas for the non-dominant group, there was a power of 0.26 with a similar effect size of 0.38. Although previous studies demonstrated the immediate positive effects of the CMIT on the paretic upper limb's unimanual capacity^33^^,^ 34, variations regarding training duration and participants' characteristics may also have influenced these different results. The effects of dominance on unimanual capacity after mCIMT should be better investigated in future studies. 

### Study limitations

There are some limitations of the present study. Since the size of the two groups differed and the values obtained at baseline, mainly for both MAL scores, were also heterogeneous, the generalization of the present findings should be taken with caution. Although there were between-group differences at the baseline MAL scores, the groups were similar regarding the Fugl-Meyer motor assessment scores and the muscle strength. For the confirmation of these results, studies with larger samples, more homogeneous groups with specific criteria are necessary regarding the interference of upper limb dominance. 

There was no stratification of the participants regarding hand dominance prior to stroke, which could have interfered with the acquisition of the gains. However, since both groups equally improved after intervention, we believe that the participants were similar regarding hand dominance prior to stroke or that possible differences did not influence the results of the present study. Further studies are recommended to examine the influence of hand dominance prior to stroke on the acquisition and maintenance of upper limb skills. 

## Conclusions

Hand dominance did not interfere with the acquisition of upper limb skills, but influenced the maintenance of the gains observed after the application of the mCIMT for individuals with chronic stroke. The participants, whose paretic upper limb was the dominant, reported improved ability in maintaining unimanual gains. The bimanual improvements were maintained for both groups regardless the upper limb dominance previous to stroke.
